# LncRNA495810 Promotes Proliferation and Migration of Hepatocellular Carcinoma Cells by Interacting with FABP5

**DOI:** 10.3390/biology13080644

**Published:** 2024-08-22

**Authors:** Haili Wu, Haiyan Yuan, Yiwei Duan, Guangjun Li, Jin’e Du, Panfeng Wang, Zhuoyu Li

**Affiliations:** 1 College of Life Science, Shanxi University, Taiyuan 030006, China; 2 Institute of Biotechnology, The Key Laboratory of Chemical Biology and Molecular Engineering of Ministry of Education, Shanxi University, Taiyuan 030006, China; 3 Shanxi Provincial Inspection and Testing Center (Shanxi Provincial Institute of Standard Metrology Technology), Taiyuan 030006, China

**Keywords:** lncRNA495810, proliferation, migration, FABP5, hepatocellular carcinoma

## Abstract

**Simple Summary:**

In this study, the expression, biological function and potential molecular mechanisms of a novel long non-coding RNA 495810 were detected in hepatocellular carcinoma cells. The results demonstrate a significant up-regulation of long non-coding RNA 495810 in hepatocellular carcinoma, and hepatocellular carcinoma patients with higher long non-coding RNA 495810 shows poorer overall survival and disease-free survival than those with lower long non-coding RNA 495810 level. Moreover, long non-coding RNA 495810 overexpression promotes the proliferation and migration, and blocks apoptosis and cell cycle arrest of hepatocellular carcinoma cells; while knock it down get the opposite results. Mechanistically, long non-coding RNA 495810 directly binds and up-regulates fatty acid binding protein 5 expression. In further, it was found that fatty acid binding protein 5 is up-regulated in hepatocellular carcinoma and associates with poor prognosis. More importantly, fatty acid binding protein 5 knockdown reverses the enhanced abilities of proliferation and metastasis induced by long non-coding RNA 495810 overexpression. These results reveal a novel mechanism of hepatocellular carcinoma metastasis guided by long non-coding RNA, providing a potential target for the treatment of liver cancer.

**Abstract:**

Hepatocellular carcinoma (HCC) is one of the malignant tumors with high morbidity and mortality. Long non-coding RNAs (lncRNAs) are frequently dysregulated in human cancers and play an important role in the initiation and progression of HCC. Here, we investigated the expression of a new reported lncRNA495810 in our previous study by analyzing the publicly available datasets and using RT-qPCR assay. The cell proliferation experiment, cell cycle and apoptosis assay, wound healing assay, cell migration assay were used to explore the biological function of lncRNA495810 in HCC. The western blot, RNA pull down and RNA immunoprecipitation (RIP) detection were used to investigate the potential molecular mechanisms of lncRNA495810. The results demonstrated that lncRNA495810 was significantly upregulated in hepatocellular carcinoma and associated with poor prognosis of hepatocellular carcinoma patients. Moreover, it proved that lncRNA495810 promotes the proliferation and metastasis of hepatoma cells by directly binding and upregulating the expression of fatty acid-binding protein 5. These results reveal the oncogenic roles of lncRNA495810 in HCC and provide a potential therapeutic target for HCC.

## 1. Introduction

Liver cancer is a common malignant tumor. In terms of the rate of incidence and mortality, it ranks sixth and third worldwide, respectively. In 2020, there were 910,000 new cases of and 830,000 deaths from liver cancer [1]. Hepatocellular carcinoma (HCC) accounts for nearly 90% of the total cases and is the major type of liver cancer [2]. The sensitivity and specificity of traditional diagnostic markers for liver cancer are not ideal, which is not conducive to the diagnosis and treatment of early HCC. Therefore, it is necessary to search for new diagnostic biomarkers and identify credible therapeutic targets.

Long non-coding RNAs (lncRNAs) are a class of RNA molecules with transcripts longer than 200 nucleotides. Generally, there is no ability to code for proteins, but some techniques can encode short peptides [3,4,5,6,7,8]. The mechanism whereby lncRNA works is extremely complicated and mostly relies on its precise sequence and secondary structure; it also regulates various aspects of the process including transcription, splicing, RNA degradation, translation, and protein stability through interacting with DNA/RNA, microRNAs, or proteins [9]. A study by Li et al. showed that one single lncRNA will generally be bound and regulated by one or multiple RNA-binding proteins (RBPs); this combination may then coordinately regulate gene expression [10].

Additionally, the function of lncRNA is closely related to its relative abundance. Numerous studies have demonstrated that lncRNA is abnormally expressed in many tumors and is involved in the processes of tumor cell proliferation, migration, invasion, angiogenesis, reprogramming of energy metabolism, and evasion of immune regulation [11]. A large number of studies have confirmed the relationship between lncRNA and HCC. A novel lncRNA RP11-386G11.10 reprograms lipid metabolism to promote hepatocellular carcinoma progression [12]. Studies have confirmed that LINC01089 is a super-enhancer-driven lncRNA, which can promote the epithelial–mesenchymal transformation (EMT), migration, invasion, and metastasis of HCC cells in vivo and in vitro [13]. LncRNA TUG1 has been found to mediate HCC cell growth and promote EMT and metastasis [14]. LncMMPA can polarize M2 macrophages and increase HCC cell proliferation by interacting with miR-548s [15]. Also, studies have shown that lncRNA SNHG5 promotes the proliferation of HCC by regulating UPF1 and the Wnt-signaling pathway [16]. LncRNA-PDPK2P was reported to promote hepatocellular carcinoma progression through the PDK1/AKT/Caspase 3 pathway [17]. As for lncRNA495810, our previous studies have identified it as a novel tumor driver in colorectal cancer, which is highly expressed in colorectal cancer (CRC) and correlated with poor prognosis in colorectal cancer patients. Furthermore, lncRNA495810 facilitates proliferation and aerobic glycolysis in CRC cells by enhancing pyruvate kinase isozyme M2 (PKM2) protein stability via the proteasomal degradation pathway [18]. However, the functions of lncRNA495810 in HCC and specific mechanisms remain largely unknown.

The current study aims to investigate the expression, biological function, and potential molecular mechanisms of lncRNA495810 in HCC. By analyzing the publicly available datasets, it was found that lncRNA495810 was upregulated in HCC and associated with a poor prognosis in HCC patients. Moreover, lncRNA495810 promoted the proliferation and metastasis of HCC cells. In addition, a positive relationship between lncRNA495810 and fatty acid-binding protein 5 (FABP5) was observed, which was identified to mediate the oncogenic roles of lncRNA495810 in HCC. We hope that this study provides a theoretical basis for the diagnosis and clinical treatment of HCC.

## 2. Materials and Methods

### 2.1. Cell Culture and Reagents

Human hepatoma HepG2 and BEL-7402 cell lines were preserved in our laboratory. HepG2 cells were cultured in a DMEM medium and BEL-7402 cells were cultured in an RPMI-1640 medium. All media contained 10% fetal bovine serum and 1% antibiotics (100 U/mL of penicillin and 100 mg/mL of streptomycin). The cells were cultured at 37 °C in an incubator with 5% CO_2_.

### 2.2. Data Sources and Bioinformatics

Data relating to lncRNA495810 expression in human HCC tissues; the correlation of lncRNA495810 expression and disease-free survival and overall survival; the correlation of lncRNA495810 expression and the pathological stage of HCC, the relationship between FABP5 expression and the stage of LIHC; and the relationship between FABP5 expression and the overall survival of HCC patients are all from the TCGA database. The level of FABP5 protein in ten cancers and the expression of FABP5 protein in HCC were obtained from the CPATC database.

### 2.3. Transfection

The cells were inoculated in a six-well plate, Lipofectamine2000 (Invitrogen, Carlsbad, CA, USA) was used to transfect lncRNA495810 overexpression plasmids (pCDH and 495810), knockdown plasmids (shNC, sh7, sh2660), or FABP5 siRNAs (GenePharma, Shanghai, China) into HCC cells for 48 h; cells were then collected for subsequent experimental analysis. The sequences of FABP5 siRNAs were as following: 5′-CACUUGUGAUGGUAAAAACTT-3′ and 5′-GUUUUUACCAUCACAAGUGTT-3′.

### 2.4. Real-Time Quantitative Polymerase Chain Reaction (RT-qPCR)

Using an All-in-one First Strand cDNA Synthesis Kit II (Seven, Beijing, China), total RNA extracted with RNAkeyTMReagent (Seven, Beijing, China) was reverse-transcribed into cDNA, and qPCR was performed using the 2×SYBR Green qPCR MasterMix II kit (Seven, Beijing, China). GAPDH acted as a reference gene for mRNA and the 2^−ΔΔCT^ method was used to calculate the relative expression level.

### 2.5. Cell Proliferation Experiment

For the colony formation experiment, 2 × 10^3^ cells were firstly inoculated in the six-well plate and transfection was followed by culture for about 2 weeks. The cells were then washed with phosphate-buffered saline (PBS), fixed in 4% PFA for 15–20 min, and stained with 0.1% crystal violet solution for 10–15 min for further analysis and photography. For the Edu experiment, cells were first inoculated into a 96-well plate with a density of 1 × 10^5^ cells per well, transfected with the corresponding plasmid, incubated in 50 μM Edu buffer (KeyGEN BioTECH, Nanjing, Jiangsu, China) at 37 °C for 2 h, fixed with 4% paraformaldehyde (PFA) for 30 min, and permeated with 0.1% Triton X-100 for 20 min. Next, Edu solution was added to the 96-well plate, the nucleus was then stained with DAPI solution (Solarbio, Beijing, China), and the results were observed using fluorescence microscopy (Nikon, Shanghai, China).

### 2.6. Apoptosis Detection

The cells were inoculated in a 6-well plate. After 48 h of experimental treatment, the cells were washed with PBS and stained with an Annexin V-FITC/PI double-staining apoptosis detection kit (Seven, Beijing, China). In the kit, Annexin V is the most sensitive indicator for detecting early cell apoptosis; PI (Propidium Iodide) is a commonly used indicator for late-stage apoptosis and dead cells. Early apoptosis should be Annexin V (+) PI (−), located in the lower right quadrant; late-stage apoptosis should be Annexin V (+) PI (+), located in the upper right quadrant. Finally, apoptotic cells were analyzed by flow cytometry.

### 2.7. Cell Cycle Assay

Cells were first inoculated in a 6-well plate. After 48 h of treatment, the cells were washed with PBS and then treated with a cell cycle detection kit (KeyGEN BioTECH, Nanjing, China). The cell cycle was detected by flow cytometry.

### 2.8. Wound Healing Assay

After the cells had been inoculated in a 12-well plate, treated, and grown to 90% confluency, a 200 μL pipette tip was applied across the confluent cell monolayer and then washed with PBS to remove the isolated cells and prepare an artificial wound. The cells were grown in a serum-free medium for 0 h, 24 h, and 48 h. A microscope was used to evaluate cell migration, and ImageJ (version 1.8.0, National Institutes of Health, Bethesda, MD, USA) was used for the analysis.

### 2.9. Cell Migration Assay

HepG2 and BEL-7402 cells were first seeded into the Transwell chambers (Corning, New York, NY, USA) using a serum-free medium (Seven, Beijing, China). The chambers were then placed in 24-well plates, and a medium containing 10% FBS (Gibco, San Diego, CA, USA) was added below the chambers. The 24-well plates were incubated at 37 °C for 48 h and then stained with 0.1% crystal violet solution for further analysis and photography.

### 2.10. Western Blot

The cells were cleaved on ice for 30 min in a lysate containing 1% phenylmethyl sulfonyl fluoride (1 mM). The protein concentration was determined using a BCA protein concentration assay kit (Seven, Beijing, China). After boiling at 95 °C for 10 min to denature the extracted protein, an equal amount of the protein was separated on the SDS-PAGE gel and transferred to the PVDF membrane. After the completion of blocking, the membranes were incubated with indicated antibodies overnight at 4 °C. On the second day, after washing with TBST, the membranes were incubated with a secondary antibody at room temperature for 1.5 h. Western blot signals were obtained from the imaging system using a hypersensitive ECL chemiluminescence detection kit (Seven, Beijing, China). GAPDH was used as a control.

### 2.11. RNA Pull-Down

The sense and antisense of lncRNA495810 were transcribed in vitro using a TranscriptAid T7 High Yield Transcription Kit (Invitrogen, Carlsbad, CA, USA). They were also biotinylated with Bio-16-UTP (Life Technology, Shanghai, China) according to the manufacturer’s instructions. The DNA template was a PCR product containing a T7 promoter sequence upstream. For the RNA pull-down assay, cells were collected and lysed on ice for 1 h. Streptavidin Agarose beads (Invitrogen, Carlsbad, CA, USA) and biotin-labeled sense or antisense of lncRNA495810 were incubated at 4 °C for 1 h, then mixed with the cracked supernatant and incubated at 4 °C overnight. Western blot detection was performed after incubation.

### 2.12. RNA Immunoprecipitation (RIP) Detection

Cells were collected from a 100 mm culture dish and lysed on ice using Western/IP cell lysis solutions (Beyotime, Shanghai, China) for 1 h. After centrifuging, a small portion of the cell lysis supernatant was obtained for Western blot analysis, while the other portion was mixed with antibodies against FABP5 or IgG overnight. Protein A + G Agarose (Beyotime, Shanghai, China) was added and allowed to mix for 2 h. Finally, RNA was extracted and the enrichment of lncRNA495810 in co-precipitated RNA was detected using RT-qPCR. IgG was used as a negative control.

### 2.13. Statistical Analysis

Statistical analyses were performed using the SPSS software (version 28.0.1.1, Norman H. Nie, Dale H. Bent, Chicago, IL, USA). Continuous variables were presented as means ± standard deviations. The differences between the two groups were verified using Student’s *t*-test. The survival analysis was based on the TCGA database and was analyzed using a Kaplan–Meier Plotter. All in vitro assays were independently repeated at least three times.

## 3. Results

### 3.1. LncRNA495810 Is Upregulated in HCC and Associated with Poor Outcomes in HCC Patients

**Figure 1:**
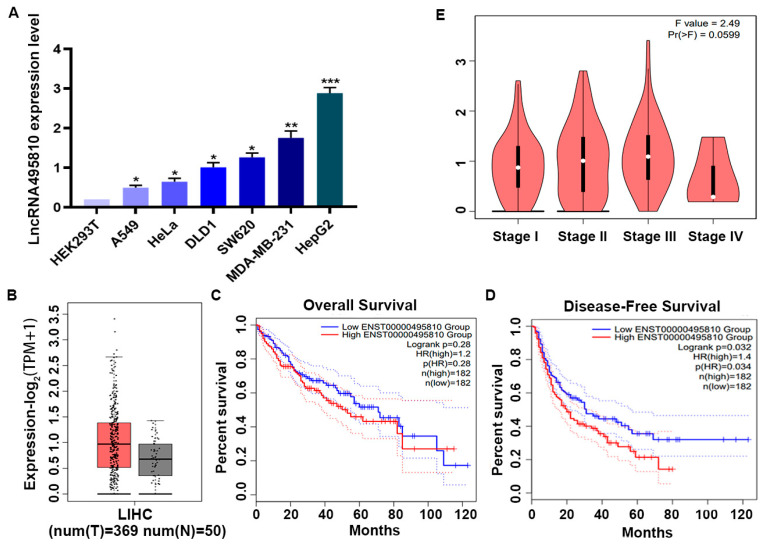
**LncRNA495810 is upregulated in HCC and associated with poor outcomes in HCC patients.** (**A**) LncRNA495810 expression level was detected by RT-qPCR in colorectal cancer, breast cancer, cervical cancer, lung cancer, and HCC cells. (**B**) LncRNA495810 expression in human HCC tissues (T, n = 369) and normal liver tissues (N, n = 50) based on TCGA data from GEPIA2. (**C**,**D**) Kaplan–Meier survival analysis of the correlation between lncRNA495810 expression and disease-free survival and overall survival based on TCGA liver cancer data, analyzed by the online in silico tool Kaplan–Meier Plotter (https://kmplot.com/analysis/, accessed on 18 March 2024). (**E**) The correlation between lncRNA495810 expression and HCC pathological stage is based on TCGA data from GEPIA2. * *p* < 0.05, ** *p* < 0.01, *** *p* < 0.001.

One of our previous studies has shown that lncRNA495810 was aberrantly highly expressed in colorectal cancer cells compared with the normal ones. Herein, the expression level of lncRNA495810 was firstly compared in different tumor cells, including colorectal cancer, breast cancer, cervical cancer, lung cancer, and HCC cells, and it was found to be significantly increased in the HCC cell line HepG2 compared to other cell lines (Figure 1A). To clarify the expression of lncRNA495810 in HCC, we then analyzed the TCGA data from GEPIA 2. The data revealed that the expression of lncRNA495810 in HCC was markedly higher than that in normal liver tissues (Figure 1B). More importantly, a survival analysis indicated that high lncRNA495810-expressing patients showed an evidently poorer disease-free survival and overall survival compared to HCC patients with low lncRNA495810 expression (Figure 1C,D). However, it was not correlated with an advanced clinical grade and stage (Figure 1E).

### 3.2. LncRNA495810 Promotes the Proliferation of HCC Cells

**Figure 2:**
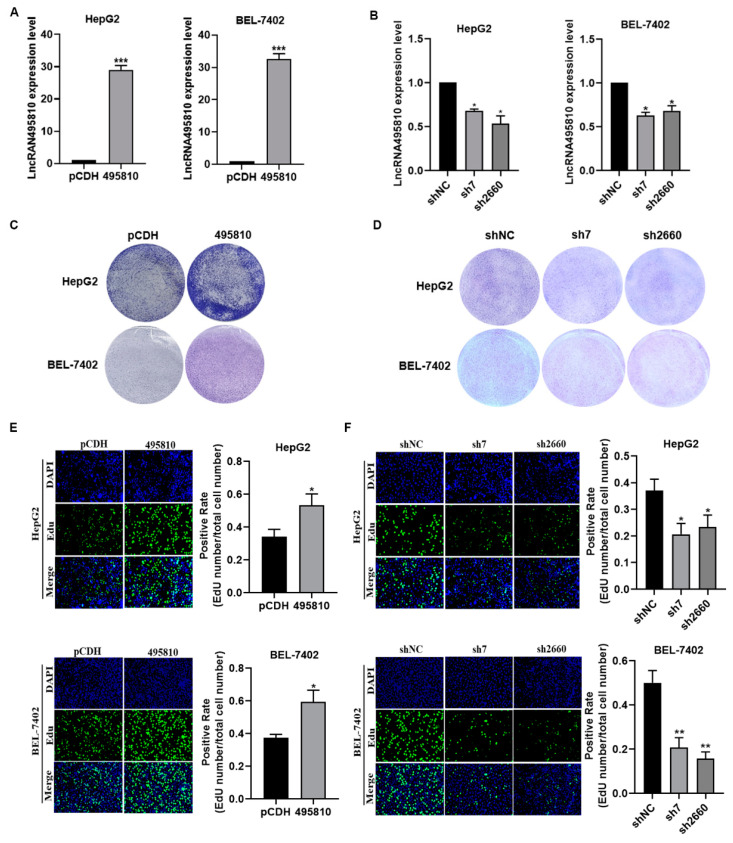
**LncRNA495810 promotes the proliferation of HCC cells.** (**A**) After the transfection of empty plasmid (pCDH) and lncRNA495810-overexpressed plasmid (495810) in HepG2 and BEL-7402 cells, the mRNA expression level of lncRNA495810 was analyzed by RT-qPCR. (**B**) RT-qPCR analysis of lncRNA495810 mRNA expression level after HCC cells were transfected with control plasmid (shNC) and lncRNA495810 knockdown plasmid (sh7, sh2660). (**C**) HepG2 and BEL-7402 cells were transfected with empty plasmid (pCDH) and lncRNA495810-overexpressed plasmid (495810) for colony formation analysis. (**D**) Colony formation analysis after HCC cells were transfected with control plasmid (shNC) and lncRNA495810 knockdown plasmid (sh7, sh2660). (**E**) Cell proliferation was analyzed by Edu assay after empty plasmid (pCDH) and lncRNA495810-overexpressed plasmid (495810). (**F**) The cell proliferation was analyzed by Edu assay after lncRNA495810 knockdown (sh7, sh2660). The proliferation ability of HepG2 cells and BEL-7402 cells after over-expression or silencing of lncRNA 495810 determined by Edu assay (×200). * *p* < 0.05, ** *p* < 0.01, *** *p* < 0.001.

To further explore the biological roles of lncRNA495810 in HCC, HepG2 and BEL-7402 cells were transfected with lncRNA495810 overexpression or knockdown plasmid, and the transfection efficiency was detected by RT-qPCR. It can be seen that lncRNA495810 expression was remarkably restored in both cells transfected with overexpressed plasmid (Figure 2A) and that it was suppressed in cells transfected with indicated shRNAs (Figure 2B). The colony formation experiment and Edu assay were then carried out to evaluate the role of lncRNA495810 in cellular proliferation. The results showed that the number of colony-forming cells and Edu-positive cells were significantly upregulated after the overexpression of lncRNA495810 (Figure 2C,D) while they were decreased in lncRNA495810 knockdown cells (Figure 2E,F). Collectively, these data demonstrated that lncRNA495810 promoted HCC cellular proliferation.

### 3.3. LncRNA495810 Blocks Apoptosis and Cell Cycle Arrest of HCC Cells

**Figure 3:**
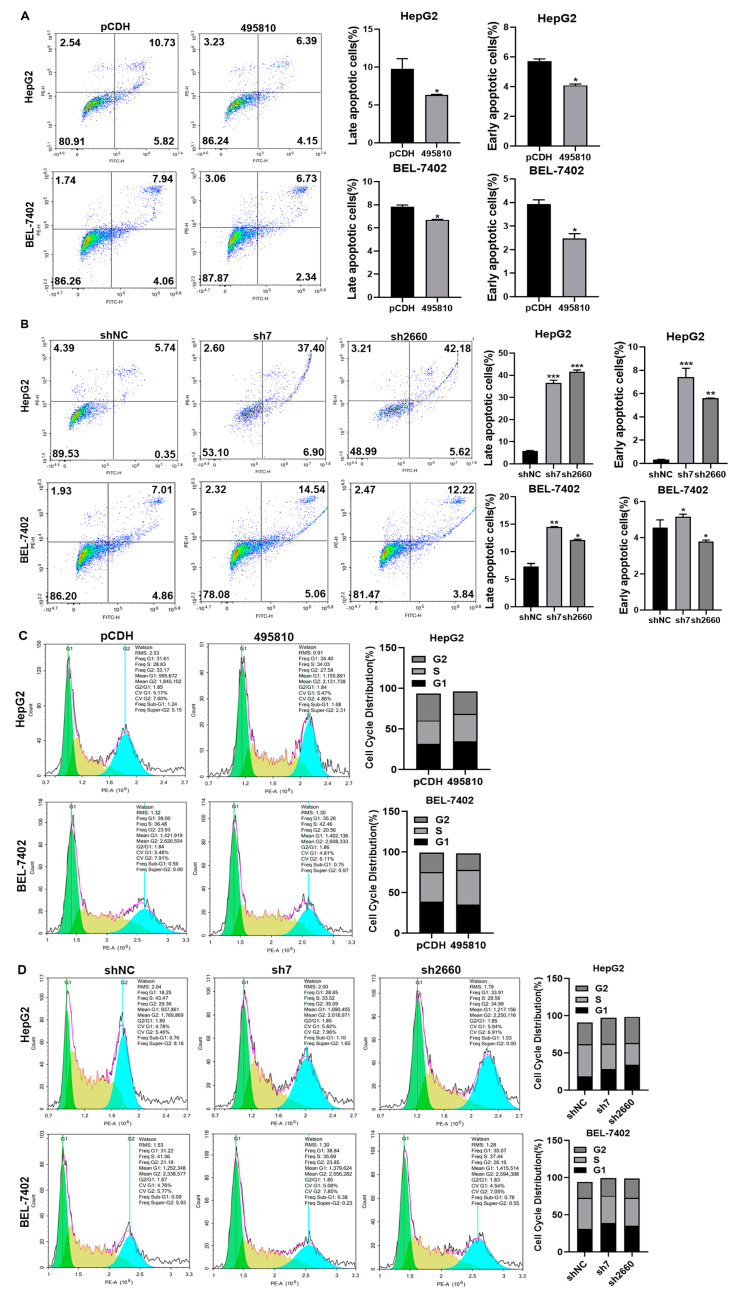
**LncRNA495810 blocks apoptosis and cell cycle arrest of HCC.** (**A**) Flow cytometry analysis of apoptosis after transfection of empty plasmid (pCDH) and lncRNA495810 overexpression plasmid (495810) in HepG2 and BEL-7402 cells. (**B**) Flow cytometry analysis of cell apoptosis after transfection of control plasmid (shNC) and lncRNA495810 knockdown plasmid (sh7, sh2660). (**C**) Flow cytometry analysis of cell cycle changes after transfection of empty plasmid (pCDH) and lncRNA495810 overexpression plasmid (495810). (**D**) Flow cytometry analysis of cell cycle changes after transfection of control plasmid (shNC) and lncRNA495810 knockdown plasmid (sh7, sh2660). * *p* < 0.05, ** *p* < 0.01, *** *p* < 0.001.

In order to investigate the effects on apoptosis and cell cycle of HCC cells, flow cytometry was used to analyze HepG2 and BEL-7402 cells overexpressing or interfering with lncRNA495810. The results showed that lncRNA495810 overexpression decreased the percentage of both late and early apoptotic cells and reduced the G1 phase arrest of cells (Figure 3A,C). In contrast, lncRNA495810 knockdown promoted the apoptosis of HepG2 and BEL-7402 cells (Figure 3B) while arresting the cell cycle in the G1 phase (Figure 3D).

### 3.4. LncRNA495810 Promotes the Migration of HCC Cells

**Figure 4:**
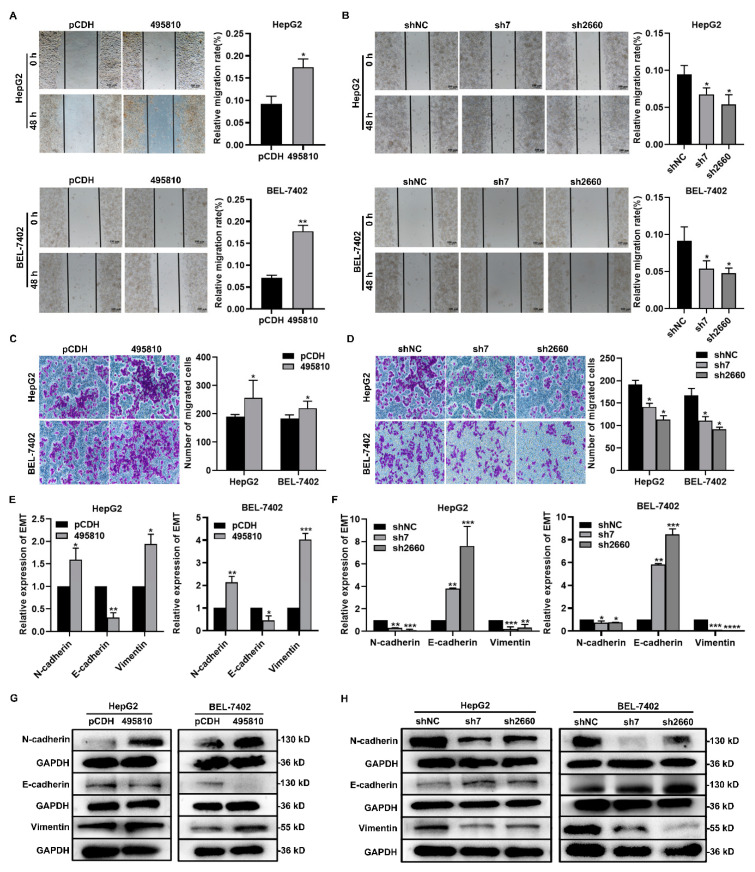
**LncRNA495810 promotes the migration of liver cancer cells**. (**A**,**C**) Wound healing assay (**A**) and migration assay (**C**) were conducted after transfection of empty plasmid (pCDH) and lncRNA495810 overexpression plasmid (495810). (**B**,**D**) Wound healing assay (**B**) and migration assay (**D**) were conducted after knockdown of lncRNA495810 (sh7, sh2660). The migration ability of HepG2 cells and BEL-7402 cells after lncRNA 495810 overexpression or silencing was determined by migration assay (×200). (**E**,**G**) The effect of lncRNA495810 on the EMT process of HCC cells was studied by RT-qPCR and Western blot after lncRNA495810 overexpression (495810). (**F**,**H**) After knocking down lncRNA495810 (sh7, sh2660), the effects of lncRNA495810 on the EMT process of HepG2 and BEL-7402 cells were studied by RT-qPCR and Western blot. * *p* < 0.05, ** *p* < 0.01, *** *p* < 0.001.

To further explore the role of lncRNA495810 in HCC progression, the wound healing assay and Transwell migration assays were carried out. The wound healing assay demonstrated that lncRNA495810 overexpression significantly facilitated the migratory capacity of HepG2 and BEL-7402 cells (Figure 4A). The Transwell assay also confirmed that after the forced upregulation of lncRNA495810, the number of migrated HCC cells was markedly increased (Figure 4C). EMT refers to the biological process by which epithelial cells are transformed into cells with a mesenchymal phenotype through a specific procedure and is considered to be a key process in the migration, invasion, and metastatic spread of cancer cells [19]. Subsequently, RT-qPCR and Western blot analyses were implemented to evaluate the expression of EMT-related proteins. It was found that lncRNA495810 overexpression significantly increased the level of mesenchymal markers, N-cadherin, and Vimentin but decreased that of the epithelial marker E-cadherin in HepG2 and BEL-7402 cells (Figure 4E,G). Meanwhile, lncRNA495810 knockdown evidently restrained the migration of HCC cells (Figure 4F,H).

### 3.5. The Expression of FABP5 Is Upregulated in HCC and Associated with Poor Prognosis

**Figure 5:**
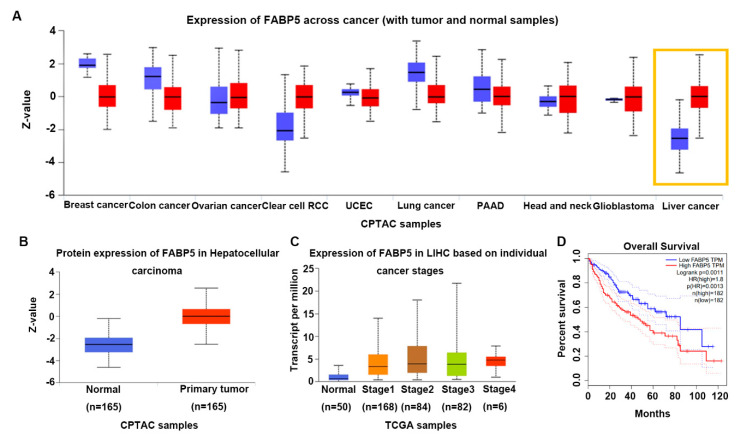
**FABP5 is upregulated in HCC and associated with a poor prognosis.** (**A**) FABP5 protein levels for ten cancers in the CPATC database. (**B**) Protein expression of FABP5 in HCC in CPATC database. (**C**) The expression of FABP5 in the TCGA database is related to the stage of LIHC. (**D**) The TCGA database showed that FABP5 expression was negatively correlated with the overall survival time of HCC patients.

In order to elucidate the underlying mechanism by which lncRNA495810 exerted oncogenic roles in HCC, we conducted RNA pull-down combined with mass spectrometry experiments to detect proteins that interact with lncRNA495810. A total of 43 proteins that specifically interact with the lncRNA495810 sense strand were detected, as listed in Supplementary Table S1, among which, fatty acid-binding protein 5 (FABP5) caught our attention. FABP5 is reported to be highly expressed in a variety of cancers and regulates the development and progression of cancer cells including HCC through its participation in the EMT signaling pathway [20]. Consistently, we found that the expression of FABP5 in HCC tissues was significantly higher than that in neighboring normal tissues by searching the UALCAN database (Figure 5A,B). But, the expression of FABP5 was found to be little related to tumor staging (Figure 5C). In addition, the Kaplan–Meier curve showed a poor prognosis in HCC patients in the FABP5-high-expression group (Figure 5D). In summary, FABP5 may be a key player involved in the HCC process.

### 3.6. LncRNA495810 Interacts with FABP5 and Is Positively Correlated with Its Expression

**Figure 6:**
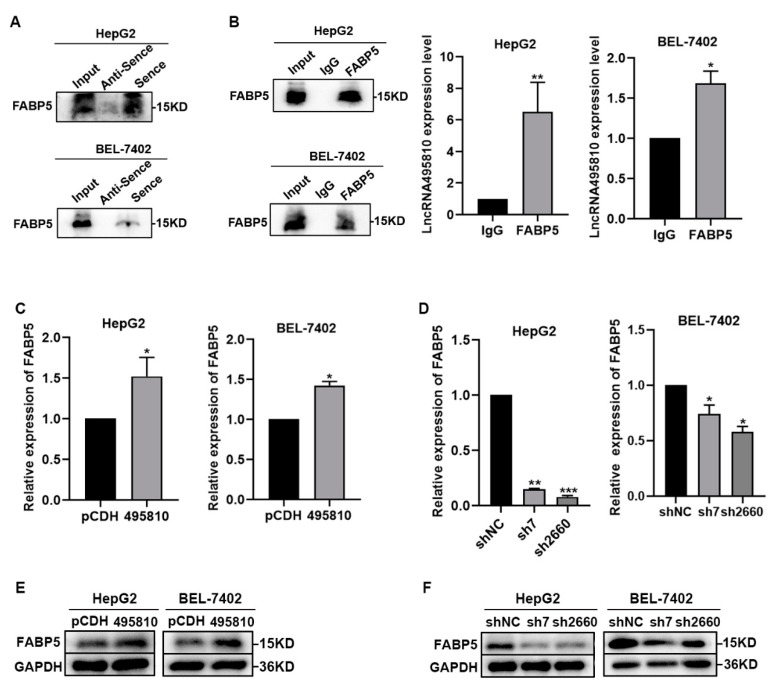
**LncRNA495810 binds to FABP5 and promotes its expression.** (**A** and **B**) RNA pull-down assay (**A**) and RIP assay (**B**) were conducted to determine the interaction between lncRNA495810 and FABP5. (**C**) RT-qPCR was performed to detect the effect of lncRNA495810 overexpression (495810) on the mRNA level of FABP5. (**D**) RT-qPCR was performed to detect the effect of lncRNA495810 knockdown (sh7, sh2660) on the mRNA level of FABP5. (**E**) Western blot was conducted to detect the effect of lncRNA495810 overexpression (495810) on FABP5 protein level. (**F**) Western blot was conducted to detect the effect of lncRNA495810 knockdown (sh7, sh2660) on FABP5 protein level. * *p* < 0.05, ** *p* < 0.01, *** *p* < 0.001.

Based on the above results, we confirmed the interaction between lncRNA495810 and FABP5 by RNA pull-down and RIP experiments (Figure 6A,B). In addition, RT-qPCR and Western blot results showed that lncRNA495810 overexpression promoted FABP5 expression (Figure 6C,E), while knocked-down lncRNA495810 inhibited the expression of FABP5 in both mRNA and protein levels (Figure 6D,F).

### 3.7. FABP5 Mediates the Promoting Effect of lncRNA495810 in HCC Cells

**Figure 7:**
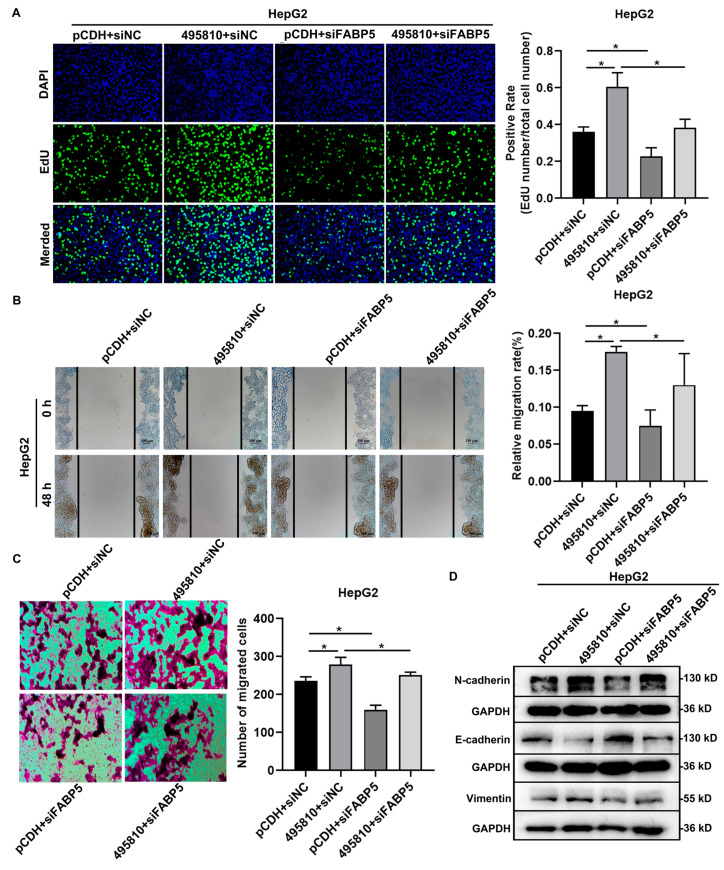
**FABP5 mediates the promoting effect of lncRNA495810 in HCC cells.** (**A**) The cell proliferation was analyzed by Edu assay after HepG2 cells were cotransfected with empty plasmid (pCDH + siNC) or lncRNA495810 overexpression plasmid (495810 + siNC) and FABP5 siRNA (siFABP5). The proliferation capacity of HepG2 cells was determined by Edu assay (×200). (**B**,**C**) The effect of cell migration was analyzed by wound healing assay (**B**) and Transwell assay (**C**) after HepG2 cells were cotransfected with empty plasmid (pCDH + siNC) or lncRNA495810 overexpression plasmid (495810 + siNC) and FABP5 siRNA (siFABP5). The migration ability of HepG2 cells was determined by migration assay (×200). (**D**) The expression of EMT-related proteins was detected using Western blot assay after HepG2 cells were cotransfected with empty plasmid (pCDH + siNC) or lncRNA495810 overexpression plasmid (495810 + siNC) and FABP5 siRNA (siFABP5). * *p* < 0.05.

To further verify the role of FABP5 in lncRNA495810 promoting the malignant progression of HCC cells, after HepG2 cells were cotransfected with lncRNA495810 overexpression plasmid and FABP5 siRNAs (siFABP5), the cell proliferation and migration ability were then detected. Edu assay indicated that siFABP5 inhibited the proliferation of HCC cells and reversed the promotion effect of lncRNA495810 overexpression (Figure 7A). Wound healing assay and Transwell assay showed that siFABP5 alone suppressed the migration, while cotransfection significantly inhibited the lncRNA495810 overexpression-induced migration ability of HepG2 cells (Figure 7B,C). Additionally, Western blot analysis showed that siFABP5 could reverse the effects of lncRNA495810 overexpression on EMT-related markers (Figure 7D). These results suggest that FABP5 at least partially mediated the oncogenic roles of lncRNA495810 in HCC.

## 4. Discussion

With the further study of lncRNAs, cancer-associated lncRNAs are emerging as one of the hottest questions in RNA biology and oncology. Recently, increasing evidence indicates that the abnormal expression of lncRNAs plays a crucial role in tumor formation and development [21,22]. In our previous studies, we found that lncRNA495810 (ENST00000495810) was highly expressed in many tumors, and it was localized to chr7: 26069506-26130778 with a length of 484 bp and two exons, which were predicted by ORF finder and CSF to have no encoding potential. Herein, the essential roles of lncRNA495810 in HCC growth and metastasis were investigated. The results demonstrated that lncRNA495810 is upregulated in HCC and associated with poor survival in HCC patients. Moreover, the high expression of lncRNA495810 significantly promoted the proliferation and migration of HCC cells, and knockdown facilitated the growth and metastasis of HCC cells. These data suggest that lncRNA495810 acts as an oncogene in HCC.

FABP5, an intracellular companion of fatty acid molecules that regulates lipid metabolism and cell growth, has been found to be abundantly expressed in different cancer types and involved in a variety of biological processes such as proliferation, differentiation, migration, and invasion [23]. For example, FABP5 was reported to promote liposolysis and fatty acid synthesis, which leads to an increase in intracellular fatty acids that activate NF-κB signaling, thereby inducing lymph node metastasis [24]. In HCC cells, by enhancing HIF-1α activity through disrupting FIH/HIF-1α interaction, FABP5 promotes lipid accumulation and cell proliferation [25]. In addition, FABP5 has also been shown to promote angiogenesis through activating the IL6/STAT3/VEGFA pathway in HCC [26]. Tang et al. identified the role of FABP5 in promoting the proliferation and migration of HCC via the CREB/miR-889-5p/KLF9 axis [27]. All of these processes indicate that FABP5 may be a potential target for the treatment of HCC. In the present study, by searching the public databases, we found a significant positive correlation between lncRNA495810 and FABP5, and more importantly, lncRNA495810 was proven to exert a promoting effect on HCC by upregulating the expression of FABP5. In line with the above results, cells transfected with FABP5 siRNA inhibited their proliferation and migration ability compared with the siNC group.

Numerous studies suggest that lncRNAs can induce a series of cancer phenotypes such as sustained proliferation, metabolic abnormalities, and metastasis by regulating the expression, localization, stability, and activity of their binding partners, leading to the tumorigenesis and progression of HCC [28]. It has been reported that lncRNA HEPFAL accelerates ferroptosis in hepatocellular carcinoma by regulating SLC7A11 ubiquitination [29]. LncRNA MIAT promotes the proliferation and invasion of HCC cells via sponging miR-214 [30]. Studies have shown that lncRNA AIRN can bind to STAT1, enhance the stability of STAT1 protein, and finally inhibit the proliferation of HCC cells and promote cell apoptosis [31]. LncRNA MRVI1-AS1 was found to accelerate hepatocellular carcinoma progression by recruiting the RNA-binding protein CELF2 to stabilize SKA1 mRNA [32]. LncRNATINCR has been reported to directly bind TCPTP and inhibit STAT3 dephosphorylation, thereby promoting STAT3 activation and the role of its downstream target genes in HCC progression and tumorigenicity [33]. It has also been proven that lncRNADBET can interact with FABP5 to activate the PPAR signaling pathway and promote lipid metabolism in cancer cells, ultimately promoting the malignant progression of bladder cancer [34]. Herein, we found that lncRNA495810 can bind to FABP5 and affect the EMT process of HCC cells by affecting the expression of FABP5. Therefore, we speculate that lncRNA495810 combined with FABP5 will affect the expression of EMT process downstream factors, but the specific process still needs to be further explored, which is also the target of our follow-up research.

## 5. Conclusions

In conclusion, this study illustrates the carcinogenic effect of lncRNA495810 in HCC cells. It also finds that a high expression of lncRNA495810 can significantly promote the proliferation and migration of HCC cells via the lncRNA495810/FABP5 axis. These results suggest that lncRNA495810 may be a potential prognostic marker and therapeutic target for HCC.

## Data Availability

All data needed to evaluate the conclusions are presented in this paper.

## Supplementary Materials

The following supporting information can be downloaded at: https://www.mdpi.com/article/10.3390/biology13080644/s1, Table S1: Proteins specifically interacted with lncRNA495810 sense strand.
